# Anosmia and COVID-19: perspectives on its association and the pathophysiological mechanisms involved

**DOI:** 10.1186/s41983-020-00266-0

**Published:** 2021-01-07

**Authors:** Paulo Roberto da Silva Júnior, André Luis Oliveira Ramos Gomes, Lucas Eugênio Araújo Coelho, Mariana Almeida Morais, Pedro Vinícius Furtado Carneiro de Almeida, Wagner José Raia Neri, Guilherme Veras Mascena, Adriana Amorim de Farias Leal

**Affiliations:** Department of Medicine, Faculty of Medical Sciences of Campina Grande, Unifacisa University Center, Senador Argemiro de Figueiredo Avenue, 1901, Campina Grande, PB 58411-020 Brazil

**Keywords:** Coronavirus infections, Olfaction disorders, Neurologic manifestations, Covid-19, SARS-CoV-2, Anosmia

## Abstract

With the spread of SARS-CoV-2, contingency measures and plans to facilitate the screening of infected patients are needed. Changes in olfaction have been cited as symptoms of the disease, and it is important to prove or exclude its association with this condition to refine the symptomatic criteria for early isolation. This article aims to analyze the association between olfactory disorders and SARS-CoV-2 infection as well as investigate the possible underlying pathophysiological mechanisms. The research was carried out using the PubMed, Science Direct, and LILACS databases on May 9, 2020, and updated on May 21. Combinations of MeSH descriptors and the Boolean operator, “AND,” were used: coronavirus infections AND olfactory disorders, coronavirus infections, and neurological manifestations. A total of 1187 articles were found in the databases, of which 17 were included in the study. The data suggest that changes in smell are strongly associated with Covid-19, especially in women and patients with fever; these changes increase the degree of suspicion of Covid-19, and they warrant early implementation of isolation and surveillance measures. There are still gaps in the elucidation of the pathways involved in the loss of smell caused by SARS-CoV-2; however, the great affinity of the virus for ACE-2 receptors, which are present in large quantities in the nasal cavity and olfactory bulb, has been considered.

## Introduction

The coronavirus disease of 2019 (Covid-19) is an infection caused by the severe acute respiratory syndrome coronavirus type 2 (SARS-CoV-2), which is characterized by respiratory failure in its most severe form of presentation. The first case was described in Wuhan, China, from where it rapidly spread to 188 countries. In March 2020, it was declared a pandemic by the World Health Organization (WHO) [1]. More than 14.5 million people have been infected since July 20, 2020, and more than 600,000 have died due to complications [2].

The diagnosis of Covid-19 is confirmed by molecular or serological exam, but the presence of symptoms such as cough, fever, chills, dyspnea, headache, muscle pain, and odynophagia increase its degree of suspicion [3]. In addition, other possible symptoms related to the condition have been reported, including anosmia and hyposmia, characterized by the total and partial absence of smell, respectively. A recent study published in the American Journal of Otolaryngology supports this hypothesis, suggesting that infection by SARS-CoV-2 may develop with different degrees of olfactory disorders (OD) [3–5]. However, there are still doubts about its clinical presentations. A meta-analysis revealed a high prevalence of olfactory symptoms in infected patients [5]. An Italian and an Iranian cohort demonstrated the loss of smell in hospitalized patients with a confirmed diagnosis. However, the association between Covid-19 and olfactory symptoms needs further clarification [6, 7].

Symptomatological understanding is essential for diagnosis and the establishment of appropriate health surveillance measures [5]. Most individuals with anosmia neglect contact precautions, as they consider themselves relatively asymptomatic or do not recognize such symptoms as manifestations of the disease [8]. Owing to its clinical relevance, the American Academy of Otorhinolaryngology proposed that ODs should be indicated for the screening of suspected patients, in addition to isolation, to prevent further spread of the virus [4, 5].

There are limited data on this topic, which is significantly attributable to the recent emergence of SARS-CoV-2. However, there has been widespread communication in the media about patients who had anosmia or hyposmia during the infection [9]. In addition, researchers have demonstrated the central nervous system (CNS) entry of the virus through the olfactory bulb in experimental models, suggesting its relationship with changes in olfactory function [10].

Thus, a review of the current medical literature was conducted to investigate the association between SARS-CoV-2 infection and the loss of smell as well as the possible underlying etiopathogenic mechanisms.

## Methods

The literature review followed the PRISMA (Preferred Reporting Items for Systematic Reviews and Meta-Analyses) 2009 criteria, which had been summarized into a 27-item checklist to guide researchers in conducting reviews [11]. A protocol was developed, based on the PICOS (Problem, Intervention, Comparison, Outcome, Study Design) strategy, to summarize the main points of the search and data extraction as well as assist in the elaboration of the guiding question of the investigation [12].

Observational studies, literature reviews, and case reports, written in Portuguese, English, or Spanish and published between January 1, 2020, and May 21, 2020, were included. The following exclusion criteria were adopted: (a) studies that did not respond to the research question, (b) articles referring to a type of coronavirus other than SARS-CoV-2, (c) papers published before 2020, (d) duplicate articles, and (e) papers published in the form of book chapters, encyclopedias, conference or conference proceedings, theses, and dissertations.

The research question was based on the association between olfactory symptoms and the SARS-CoV-2 infection and the possible underlying pathophysiological mechanisms. A literature investigation was carried out on May 9, 2020, with subsequent updates on May 21, using the PubMed, Science Direct, and LILACS databases.

For this purpose, MeSH descriptors were used, and they were combined with the “AND” and “OR” Boolean operators: “Coronavirus Infections” AND “Olfaction Disorders” OR “Coronavirus Infections” AND “Neurologic Manifestations”.

The number of Covid-19 patients who presented with olfactory symptoms was analyzed as a quantitative variable and presented as relative frequency. The pathophysiology of the virus in the central nervous system and the association between olfactory disorders and SARS-CoV-2 infection were analyzed as qualitative variables.

## Results

As shown in Table 1, after searching the PubMed database using the combination of MeSH descriptors, 105 articles were found; of these, 38 were published before 2020, 47 did not comply with the topic of interest of this study, 2 did not satisfy the language criteria, and 1 was an editorial, and they were excluded. With the same descriptors, 1081 papers were obtained from Science Direct; of these, 921 were published before 2020, 90 did not comply with the topic of interest of this study, and 2 were published in a language other than Portuguese, English, or Spanish, and they were excluded.

**Table 1:** Description of search results from the databases

Association between descriptors	PubMed	Science Direct	LILACS
“Coronavirus Infections” AND “Olfaction Disorders”	10	43	01
“Coronavirus Infections” AND “Neurologic Manifestations”	95	1038	0
Total	105	1081	01

Adapted from Aguiar and colleagues [13] and Oliveira and colleagues [14]

In addition, 57 papers did not meet the inclusion criteria because they were published as book chapters, encyclopedias, conference or conference proceedings, theses, or dissertations. Only 1 article was found from the LILACS database and included.

**Fig. 1:**
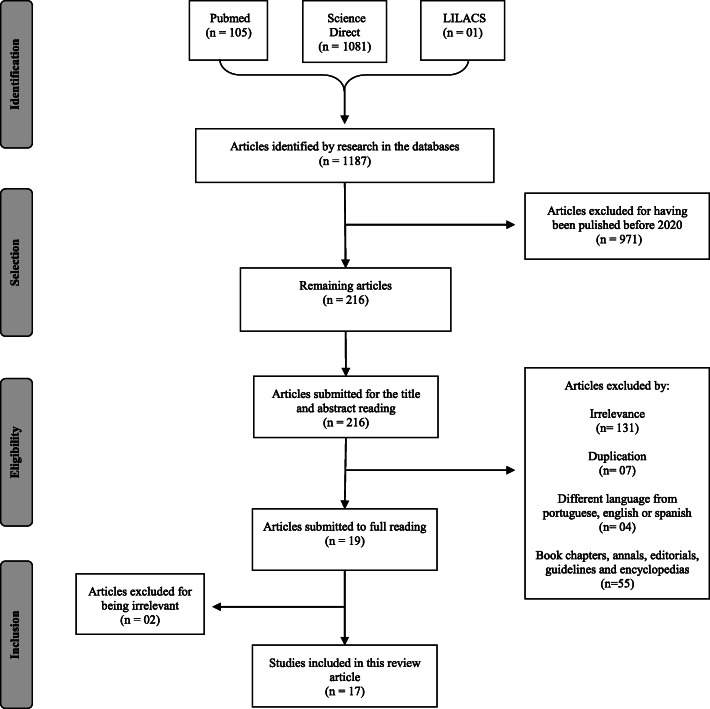
Flow diagram for the study selection process based on the inclusion and exclusion criteria. Adapted from Galvão and colleagues [11]

In general, 1187 articles were found in the databases, of which 971 articles were excluded as they were not published in 2020. After reading the titles and abstracts of the remaining 216 articles, some were further excluded; 131 articles did not satisfy the study scope, 7 were duplicated, 4 were written in different languages, and 55 were neither review articles, observational studies, nor case reports (Fig. 1).

The articles classified as potentially relevant were fully reviewed by two independent reviewers. For the case of divergence in accepting or excluding a paper, a final decision was based on the third revision. Of the 19 studies fully read, 17 were included and 2 were excluded because they were irrelevant to the scope of this article.

As described in Table 2, among the 17 articles included in the study, 3 (17.6%) were observational cohort studies, 2 (11.76%) were cross-sectional epidemiological studies, 1 (5.88%) was a systematic review and meta-analysis, and 11 (64.70%) were literature reviews. The majority of studies were carried out in European countries or the United States of America (USA): 9 (52.94%) studies were carried out in European countries, followed by 4 (23.52%) in the USA, 2 (11.76%) in the USA and Iran, 1 (5.88%) in Pakistan, and 1 (5.88%) in Colombia.

**Table 2:** Description of the characteristics of the articles included in the study

Reference	Month/year of publication	Study type	Country	Institution	Scientific Journal	Sample (***n***)
[15]	April, 2020	Literature review	Switzerland	Geneva Centre for Emerging Virus Diseases	The British Medical Journal	-
[16]	April, 2020	Literature review	Germany	Springer Verlag GmbH	European Archives of Otorhinolaryngology	-
[17]	April, 2020	Literature review	Italy	Sapienza University of Rome	European Review for Medical and Pharmac. Sciences	-
[18]	May, 2020	Observational Cohort	England	Multicenter Study	Journal of Otolaryngology - Head & Neck Surgery	382
[19]	April, 2020	Cross-sectional epidemiological study	Italy +3 European counties	Multicenter Study	European Archives of Otorhinolaryngology	417
[6]	March, 2020	Cross-sectional epidemiological study	Italy	Universidade de Milan	Clinical Infectious Diseases	59
[20]	April, 2020	Observational Cohort	Netherlands	Multicenter Study	Euro Surveillance	269
[21]	April, 2020	Literature review	USA	University of California	Brain, Behavior, and Immunity	-
[8]	April, 2020	Literature review	USA	Medical University of South California	World Journal of Otolaryngology - Head & Neck Surgery	-
[22]	April, 2020	Literature review	Spain	Hospital del Mar	Acta Otorrinolaringológica Española	-
[7]	May, 2020	Literature review	Pakistan	Bahria University, Medical and Dental College	Journal of Clinical Neuroscience	-
[1]	May, 2020	Literature review	USA	Brooke Army Medical Center	American Journal of Emergency Medicine	-
[23]	May, 2020	Literature review	Iran and USA	Multicenter Study	Clinical Imaging	-
[24]	April, 2020	Literature review	Colombia	Universidad de Manizalas e Universidad de Caldas	International Journal of Odontostomatology	-
[10]	May, 2020	Literature review	Germany	TU Dresden	Journal of the Amarican Medical Association	-
[25]	April, 2020	Systematic Review and Meta-analysis	USA	School of Medicine at Hofstra/Northwell	American Academy of Otolaryngology–Head and Neck Surgery Foundation	1627
[26]	April, 2020	Observational Cohort	Iran and USA	Multicenter Study	International Forum of Allergy and Rhinology	120

Adapted from Aguiar and colleagues [13] and Johnson and colleagues [27]

*USA* United States of America

## Discussion

This review included studies that addressed olfactory changes as a possible consequence of SARS-CoV-2 infection. After a critical analysis of these articles, it was observed that the sudden onset of anosmia or hyposmia may be indicative of Covid-19, and they may warrant the implementation of early isolation measures. Table 3 shows the main results of the studies included in this review.

**Table 3:** Summary of the main results obtained from the articles included in the study

Reference	Article title	Main results
[15]	Clinical features of covid-19.	Olfactory disorders were observed in 53% of patients in a cohort study conducted in Italy.
[16]	Possible link between anosmia and COVID-19: sniffing out the truth.	Loss of smell and/or taste can be a consistent symptom of SARS-CoV-2 infection. In addition, nasal epithelial cells exhibit a very high expression of the ACE-2 receptor allowing the viral entry.
[17]	Defining the burden of olfactory dysfunction in COVID-19 patients.	The Department of Diseases at Hospital Luigi Sacco, in Milan, Italy, through a questionnaire with 59 patients hospitalized for COVID-19, found that approximately 35% of patients had olfactory or gustatory changes and 18.6% had both.
[18]	Early recovery following new onset anosmia during the COVID-19 pandemic - an observational cohort study.	Of 382 patients, 86.4% reported anosmia. Of these, 11.5% reported severe loss of smell. After a week of follow-up: 80.1% reported a decrease in the severity of the symptom, 17.1% reported no change and 1.9% worsened. After a new survey (one week later): 11.5% had achieved complete symptomatic resolution and 17.3% reported the persistence of the symptom for one to four weeks. There was a 79% recovery rate in the interval between searches.
[19]	Olfactory and gustatory dysfunctions as a clinical presentation of mild-to-moderate forms of the coronavirus disease (COVID-19): a multicenter European study.	Among 417 patients with mild to moderate COVID-19 infection, 357 (85.6%) reported olfactory disorders and 79.6% had anosmia.
[6]	Self-reported Olfactory and Taste Disorders in Patients With Severe Acute Respiratory Coronavirus 2 Infection: A Cross-sectional Study.	Among 59 patients, 20 (33.9%) reported at least one taste or olfactory disorder and 11 (18.6%) reported both.
[20]	Strong associations and moderate predictive value of early symptoms for SARS-CoV-2 test positivity among healthcare workers, the Netherlands, March 2020.	Anosmia was reported by 47% of those affected by SARS-CoV-2 and was strongly associated with positivity for SARS-CoV-2.
[21]	Are we facing a crashing wave of neuropsychiatric sequelae of COVID-19? Neuropsychiatric symptoms and potential immunologic mechanisms.	Olfactory epithelial cells express the ACE2 receptor, but the exact pathophysiology pathway of the anosmia in COVID-19 remains uncertain.
[8]	Anosmia, hyposmia, and dysgeusia as indicators for positive SARS-CoV-2 infection.	Anosmia has been expressed as a symptom in patients positive for SARS-CoV-2, ranging from 15% to 66% depending on the study.
[22]	Alteraciones Del Olfato En El Covid-19, Revisión De La Evidencia E Implicaciones En El Manejo De La Pandemia.	The authors reported that 85.6% (357/417) of patients with COVID-19 had olfactory changes, 68% in the form of anosmia and 18% with hyposmia. 11.8% of the patients had changes in their sense of smell before the onset of other symptoms.
[7]	Neurological manifestations and complications of COVID-19: A literature review.	An Iranian cohort found that anosmia and hyposmia were reported in 48.23% of the patients infected by the SARS-CoV-2. Among them, the onset of anosmia was sudden in 76.24%. However, a Chinese cohort reported impaired sense of smell in only 11 (5.1%) patients.
[1]	Neurologic complications of COVID-19.	Among patients hospitalized with COVID-19, neurological complications ranged from 6% to 36%. It is suggested that SARS-CoV-2 acts in a retrograde way along the olfactory nerve and olfactory bulb, which act as a bridge between the nasal epithelium and the central nervous system, which may explain anosmia.
[23]	Extrapulmonary manifestations of COVID-19: Radiologic and clinical overview.	Suggests that the cerebral involvement of SARS-CoV-2 occurs via the cribriform plaque by interaction with ACE2 receptors, which can lead to symptoms such as hyposmia or anosmia.
[24]	El COVID-19 también Afecta el Sistema Nervioso por una de sus Compuertas: El Órgano Vascular de la Lámina Terminal y el Nervio Olfatorio. Alerta Neurológica, Prueba de Disosmia o Anosmia Puede Ayudar a Un Diagnóstico Rápido.	It proposes that the neuroinvasive properties of COVID-19 are related to the interaction of the virus with the ACE2 receptor. Therefore, those who have an altered response to smell should be considered as suspect patients.
[10]	Olfactory Dysfunction in COVID-19.	It was observed in an Iranian study that 59 of the 60 patients hospitalized with COVID-19 had impaired smell. In a study in Italy, 64% of 202 mildly symptomatic patients reported olfaction deficiency.
[25]	The Prevalence of Olfactory and Gustatory Dysfunction in COVID-19 Patients: A Systematic Review and Meta-analysis.	Ten studies were analyzed for olfactory dysfunction (n = 1627), showing a prevalence of 52.73% among patients with COVID-19. It has been demonstrated that the use of validated methods of olfactory function considerably increases the detection of smell changes.
[26]	Smell Dysfunction: a biomarker for COVID-19.	It was observed that 98% of the 60 patients affected by COVID-19 exhibited some olfactory dysfunction. Of the 60 patients evaluated, 35 (58%) were anosmatic and only one had a normosmia (1/60; 2%). The other patients presented hyposmia.

Adapted from Silva Júnior and colleagues [28]

*COVID-19* Coronavirus disease of 2019, *SARS-CoV-2* Severe acute respiratory syndrome coronavirus type 2, *ACE-2* Angiotensin-converting enzyme 2

### The association between SARS-CoV-2 infection and the loss of smell

Tong and colleagues [25], in carrying out a systematic review and meta-analysis, evaluated 10 studies and 1627 patients. A total of 845 patients (51.9%) had varying degrees of olfactory dysfunction, with prevalence ranging from 5.14 to 98.33%, depending on the study, and an average of 52.73% (95% CI, 29.64–75.23%).

A European multicenter study evaluated 417 patients with confirmed SARS-CoV-2 infection. Of these, 357 patients (85.6%) had olfactory disorders: 284 (79.6%) had anosmia and 73 (20.4%) had hyposmia. Fever was closely associated with anosmia (*p* < 0.014), and nasal obstruction or rhinorrhea had no significant association with decreased smell [19].

Iranian researchers conducted an observational cohort study involving 120 patients (60 infected by the virus and 60 uninfected) who underwent the assessment of olfactory functions using a validated test (University of Pennsylvania Smell Identification Test (UPSIT)). Of the infected patients, 59 (98.3%) had some degree of loss of smell. Anosmia was present in 15 (25%), hyposmia of different degrees was reported by 44 (73.3%), and only 1 patient (1.7%) did not present any change. Among the controls, 49 (81.7%) had no impairment of smell, and 11 (18.3%) presented some degree of hyposmia [26].

In contrast, after evaluating 59 patients, Giacomelli and colleagues [6] observed that 20 (33.9%) had at least 1 olfactory or gustatory symptom. However, when assessing dysosmia alone, no patient reported anosmia, and only 3 (5.1%) reported hyposmia. These findings may be attributable to the small sample of the study (*n* = 59) and the self-reported patient data not supported by validated testing of olfactory function, with a possible underestimation of the loss of smell, as suggested by Tong and colleagues [25].

In the Netherlands, 803 health professionals who had mild Covid-19 symptoms were assessed using a questionnaire for general, respiratory, and/or gastrointestinal symptoms. Of these, 269 patients were asked about anosmia. Of the 79 patients who tested positive for SARS-CoV-2, 37 reported anosmia. Among the 190 patients who tested negative for the virus, only 7 reported loss of smell (OR 23.0, 8.2–64.8; *p* < 0.001) [20].

When evaluated separately, studies that analyzed patients using validated olfactory assessment instruments showed a higher prevalence of smell disorders than those that did not [25]. That may explain the large discrepancy in the prevalence of olfaction disorders among COVID-19 patients in the analyzed studies.

The researchers found that anosmia is strongly associated with test positivity, with a sensitivity of 91.2%, specificity of 55.6%, positive predictive value of 16.9%, and negative predictive value of 98.5% [20]. However, it is important to emphasize that anosmia in isolation does not necessarily confirm the diagnosis of SARS-CoV-2 infection, and it should only serve as an indication for testing and early isolation of suspected patients.

During the analysis of the articles, we verified that the majority of individuals who presented with olfactory dysfunction were females [6, 18, 19]. On the other hand, Moein and colleagues [26] observed that there was no sex-related statistical difference in the prevalence of anosmia although their sample was composed of more men than women.

According to LAO and colleagues [8], anosmia has generally been expressed as a symptom of patients who test positive for SARS-CoV-2, varying from 15 to 66% depending on the study. The loss of smell was self-limiting, more prevalent in young patients, and resolved within an average of two weeks [10].

### Possible physiopathological mechanisms involved

SARS-CoV-2 infects humans by binding to angiotensin-converting enzyme 2 (ACE-2) receptors, and certain organs seem to express these receptors more than others [29]. The pathophysiological mechanism underlying the occurrence of ODs is still not well-understood, but two explanations have been proposed.

The first hypothesis suggests peripheral viral involvement. Zou and colleagues [30] found that the greater expression of ACE-2 receptors in the cells of the nasal epithelium is responsible for the olfactory repercussions reported by patients. The binding of the virus to these receptors causes degeneration of the epithelial cells of the nasal mucosa and subsequent inflammation and damage to the neural receptors responsible for olfaction [8, 10, 17].

Another hypothesis, currently the most widely accepted, suggests the direct changes to the central nervous system by the virus. This theory is supported by an experiment carried out on mice, which were artificially infected with SARS-CoV. It was found that the virus entered the CNS structures; its first access was the nasal epithelium, and it ascended through the cribriform lamina and olfactory bulb and followed the olfactory nerve pathway, to cause anosmia or hyposmia. Given its structural similarity to SARS-CoV, it is assumed that SARS-CoV-2, in an analogous way, follows the same pathophysiological pathway in Covid-19 [31].

Corroborating the data presented above, 3 case reports, and 1 case series, changes in the olfactory bulb were detected in 8 patients diagnosed with Covid-19 using magnetic resonance imaging of the brain. Of these 8 patients, 6 had anosmia, whereas 2 did not have any olfactory dysfunction [32–35]. In contrast, microscopic changes in the bulb and olfactory tract were not observed in 18 patients affected by Covid-19, and they were submitted to autopsy [36]. However, the authors did not clarify whether patients presented anosmia during the natural history of the disease.

## Conclusions

Despite the methodological differences between the studies analyzed, this review found that SARS-CoV-2 infection is strongly associated with the development of anosmia or hyposmia, especially in females and those with fever. Although it is not a replacement for the serological and molecular tests used in the diagnosis of Covid-19, observing olfactory symptoms increases the degree of suspicion of the disease, making it possible for isolation and surveillance measures to be implemented early. It is also important that patients are informed about the transient nature of the condition.

Because of its high prevalence in carriers of the virus, public awareness should be created on sudden olfactory dysfunction as a potential symptom of Covid-19 to facilitate early detection of the disease. Outpatient facilities and hospitals should also investigate sudden olfactory dysfunction, whenever possible, using methods validated and established by scientific academies such as the UPSIT.

From a pathophysiological perspective, there are gaps in the elucidation of the pathways involved in the loss of smell by SARS-CoV-2, but the affinity of the virus for ACE-2 receptors, which are present in large quantities in the nasal cavity and olfactory bulb, has been considered.

## Data Availability

The datasets supporting the conclusions of this article are available in the Figshare repository, https://figshare.com/s/348712db5df236fd7478

## Abbreviations

COVID-19: Coronavirus disease of 2019
SARS-CoV-2: Severe acute respiratory syndrome coronavirus type 2
WHO: World Health Organization
OD: Olfactory disorders
CNS: Central nervous system
PICOS: Problem, Intervention, Comparison, Outcome, Study Design
PRISMA: Preferred Reporting Items for Systematic Reviews and Meta-Analyses
MeSH: Medical Subject Headings
UPSIT: University of Pennsylvania Smell Identification Test
OR: Odds ratio
PPV: Positive predictive value
NPV: Negative predictive value
ACE-2: Angiotensin-converting enzyme 2
USA: United States of America
